# Motorcycle injuries: a systematic review for forensic evaluation

**DOI:** 10.1007/s00414-024-03250-y

**Published:** 2024-05-20

**Authors:** Elena Giovannini, Simone Santelli, Guido Pelletti, Maria Paola Bonasoni, Elena Lacchè, Susi Pelotti, Paolo Fais

**Affiliations:** 1 Department of Medical and Surgical Sciences, Unit of Legal Medicine, University of Bologna, Via Irnerio 49, Bologna, 40126 Italy; 2 Pathology Unit, Azienda USL-IRCCS di Reggio Emilia, Via Amendola 2, Reggio Emilia, 42122 Italy

**Keywords:** Motorcycle injuries, Forensic pathology, Accident dynamics, Helmet, Driver, Traffic accidents

## Abstract

The intricate interplay of exposure and speed leave motorcyclists vulnerable, leading to high mortality rates. During the collision, the driver and the passenger are usually projected away from the motorcycle, with variable trajectories or final positions. Injuries resulting from the crash can exhibit distinct and specific characteristics depending on the circumstances of the occurrence.

The aim of this study is to provide a systematic review of the literature on injuries sustained by motorcyclists involved in road accidents describing and analyzing elements that are useful for forensic assessment.

The literature search was performed using PubMed, Scopus and Web of Science from January 1970 to June 2023. Eligible studies have investigated issues of interest to forensic medicine about during traffic accidents involving motorcycle. A total of 142 studies met the inclusion criteria and were classified and analyzed based on the anatomical regions of the body affected (head, neck, thoraco-abdominal, pelvis, and limb injuries). Moreover, also the strategies for preventing lesions and assessing injuries in the reconstruction of motorcycle accidents were examined and discussed.

This review highlights that, beyond injuries commonly associated with motorcycle accidents, such as head injuries, there are also unique lesions linked to the specific dynamics of accidents. These include factors like the seating position of the passenger or impact with the helmet or motorbike components. The forensic assessment of injury distribution could serve as support in reconstructing the sequence of events leading to the crash and defining the cause of death in trauma fatalities.

## Introduction

Road crashes highly contribute to morbidity and mortality in developed and developing nations alike. Despite motorcycles account for a small fraction of the overall circulating vehicles, they are exaggeratedly involved in road collisions, thereby carrying significant forensic and medical implications [1, 2]. The intricate interplay of exposure and speed leave motorcyclists vulnerable resulting in high mortality rates. While enclosed passenger vehicles can dissipate a notable amount of energy in a collision, somehow providing driver protection against direct impact forces, motorcyclists have less protections and are easily injured [3–5]. In traffic accidents involving motorcycles, it is essential not only to determine the cause of death but also to provide a detailed description of all injuries. This information is crucial for accurately reconstructing the sequence of events [6]. Forensic pathologists can offer some insights for collision accident responsibility by studying the injury patterns and the location of the motorcycle victim’s body [7], with implications in the criminal, civil, and insurance fields. During the collision, the driver and the passenger are usually projected away from the motorcycle, resulting in variable trajectories and final positions of difficult interpretation [8]. Therefore, the reconstruction of motorcycle traffic accidents is still a challenging task [9, 10].

Injuries resulting from a traffic accident involving motorcyclists can show distinct and specific characteristics depending on the manner of occurrence. These findings can be integrated with other available information in the investigation, including circumstantial and engineering data, to assist in the reconstruction of the dynamics of the traffic accident.

A systematic review of studies reporting injuries observed in motorcyclists involved in traffic accidents was conducted to describe and analyze elements pertinent to forensic assessment. This includes features of reported injuries, their role in causing death, and their relevance in reconstructing the dynamics of the accidents.

## Materials and methods

An electronic search was performed in 3 databases: PubMed, Scopus and Web of Science. Keywords related to the study aim and included in the search string were: (motorcycle OR motorbike) AND (traffic accident OR road traffic OR motor vehicle OR crash) AND (injury OR wound OR prevention). The Preferred Reporting Items for Systematic reviews and Meta-analyses (PRISMA) guidelines were used [11].

The English language and time interval of publication, from January 1970 to June 2023, were applied as filters. All studies (original article, case report, retrospective and prospective case series) that investigate the characteristics of injuries produced during traffic accidents involving motorcycles were included. A lot of peer-reviewed papers concerning injuries and safety measures can also be found in conference proceedings (i.e. STAPP and IRCOBI). However, for the purpose of the present review, peer-reviewed papers from conferences and proceedings were not included.

The following studies were included: (a) forensic studies involving the autopsy of victims; (b) clinical studies conducted in both living and deceased subjects within clinical settings, providing information on the distribution and injury mechanisms of injuries; (c) and traffic engineering and public health studies that analyzed strategies and technologies for injury prevention in motorcycle-related road traffic accidents.

Titles, abstracts, and full texts were screened for inclusion criteria and examined. References of the selected articles were further screened, and related papers were included as a source of additional data. The following details were collected: first authors’ names, article titles, journal names, publication years, article types (prospective studies, retrospective studies, case reports, or original articles), the number of cases, whether the autopsy had been performed and injury localization and trauma mechanisms.

## Results

**Fig. 1:**
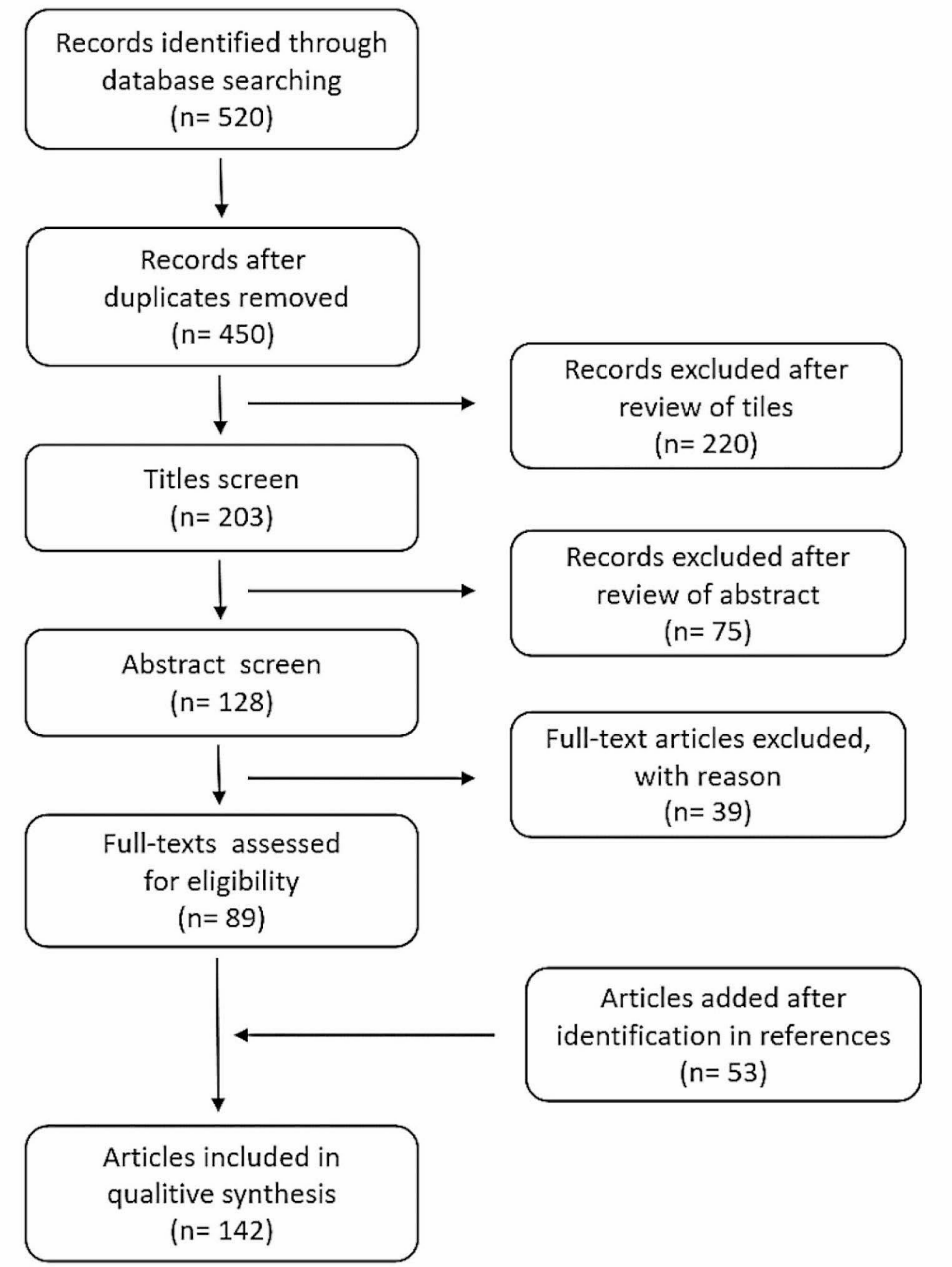
PRISMA flowchart of the present review

The results of the literature search are summarized in Fig. 1. One hundred and forty-two studies met the inclusion criteria and were included in the review. The results of the systematic review are summarized in Table 1.

**Table 1:** Studies included in the review

Author	Year	Study type	Number of cases	Autopsy performed	Article issue
Zettas et al. [12]	1979	Retrospective study	260	No	Chest, limbs injuries
Krantz et al. [13]	1985	Case report	5	Yes	Head, neck injuries
Hoekstra et al. [14]	1985	Case report	2	Yes	Head, neck injuries
Cooter et al. [15]	1988	Case report	1	Yes	Head injuries
Shiono et al. [16]	1990	Case report	1	Yes	Pelvis injuries
Shankar et al. [17]	1992	Retrospective study	1900	No	Head, neck injuries
Braddock et al. [18]	1992	Retrospective study	112	No	Head, neck, chest, abdomen, limbs injuries
Muelleman et al. [19]	1992	Retrospective study	671	No	Head injuries
Peek et al. [20]	1994	Retrospective study	700	No	Limbs injuries
Sharma et al. [21]	1995	Case report	1	Yes	Head, neck injuries
McLean et al. [22]	1995	Prospective study	14	Yes	Head injuries
Johnson et al. [23]	1995	Retrospective study	331	No	Head injuries
Tracy et al. [24]	1996	Case report	1	Yes	Abdomen injuries
Konrad et al. [25]	1996	Retrospective study	112	Yes	Head, chest, abdomen injuries
Peek-Asa et al. [26]	1996	Retrospective study	3678	No	Head, neck, chest, abdomen, limbs injuries
Wick et al. [27]	1998	Prospective study	86	No	Head, neck, chest, abdomen, pelvis, limbs injuries
Hitosugi et al. [28]	1999	Retrospective study	51	Yes	Head, neck, chest njuries
Wyatt et al. [29]	1999	Retrospective study	59	Yes	Head, neck, chest, abdomen injuries
Waikakul et al. [30]	1999	Case report	1	No	Limbs injuries
Richter et al. [31]	2001	Retrospective study	226	No	Head, neck injuries
Hitosugi et al. [32]	2001	Case report	1	Yes	Head, neck, chest injuries
Kraus et al. [33]	2002	Retrospective study	5790	No	Head injuries
Ankarath et al. [34]	2002	Retrospective study	1239	No	Head, neck, chest, abdomen, limbs injuries
Wladis et al. [35]	2002	Retrospective study	8927	No	Head, neck injuries
Lateef et al. [36]	2002	Retrospective study	1809	No	Head, neck, chest, abdomen, pelvis, limbs injuries
Watanabe et al. [37]	2002	Case report	1	Yes	Chest, pelvis injuries
Colburn et al. [38]	2003	Retrospective study	1787	No	Head, neck, chest, pelvis, limbs injuries
Kasantikul et al. [39]	2003	Prospective study	73	Yes	Head, neck injuries
Kraus et al. [40]	2003	Retrospective study	4762	No	Head, chest, abdomen, limbs injuries
Vega et al. [41]	2004	Retrospective study	29	Yes	Head, chest injuries
Hitosugi et al. [42]	2004	Retrospective study	36	Yes	Head, neck, chest injuries
Jeffers et al. [43]	2004	Retrospective study	53	No	Pelvis, limbs injuries
Grange et al. [3]	2004	Retrospective study	751	No	Neck, chest, pelvis, limbs injuries
Guerrero et al. [44]	2005	Case report	1	No	Abdomen injuries
Oliva et al. [45]	2005	Case report	1	Yes	Neck injuries
Ihama et al. [46]	2006	Case report	1	Yes	Head, pelvis injuries
Bohmer et al. [47]	2006	Case report	1	No	Abdomen injuries
Solagberu et al. [48]	2006	Prospective study	92	No	Head, neck, chest, abdomen, pelvis, limbs injuries
Zargar et al. [49]	2006	Prospective study	1332	No	Head, neck injuries
Agnihotri et al. [50]	2006	Retrospective study	217	No	Head, neck injuries
Suri et al. [51]	2007	Retrospective study	24	No	Limbs injuries
Ihama et al. [52]	2007	Case report	2	Yes	Head, neck, pelvis injuries
Munkholm et al. [53]	2007	Retrospective study	25	Yes	Head, chest, abdomen, limbs injuries
Doichinov et al. [54]	2007	Case report	1	Yes	Head injuries
Ihama et al. [10]	2008	Case report	1	Yes	Head, neck injuries
Kuo et al. [55]	2008	Case report	1	No	Head, neck, chest injuries
Robertson et al. [56]	2008	Retrospective study	39	No	Head, chest, abdomen, limbs injuries
Alicioğlu et al. [57]	2008	Retrospective study	212	No	Head, neck injuries
Bener et al. [58]	2009	Retrospective study	490	No	Head, neck, chest, abdomen, limbs injuries
Kosola et al. [59]	2009	Retrospective study	222	No	Head, limbs injuries
Mohammadi et al. [60]	2009	Retrospective study	567	Yes	Head, limbs injuries
Yeh et al. [61]	2009	Case report	1	No	Neck injuries
Murphy et al. [62]	2009	Retrospective study	42	No	Chest, pelvis, limbs injuries
Fitzharris et al. [63]	2009	Retrospective study	378	No	Head, chest, abdomen, limbs injuries
Kannus et al. [64]	2009	Experimental study	/	No	Injury prevention strategies and technologies
Talving et al. [65]	2010	Retrospective study	6530	No	Head, chest, abdomen, limbs injuries
Chalya et al. [66]	2010	Retrospective study	384	No	Head, neck, chest, abdomen, pelvis, limbs injuries
Zoja et al. [67]	2011	Case report	1	Yes	Head, neck injuries
Ogunlusi et al. [68]	2011	Prospective study	133	No	Head, neck, chest, abdomen, pelvis, limbs injuries
Amin et al. [69]	2011	Retrospective study	151	No	Head, neck, chest, abdomen, limbs injuries
Brandimarti et al. [70]	2011	Case report	2	Yes	Chest injuries
Zhao et al. [71]	2011	Retrospective study	84	Yes	Head, chest, abdomen injuries
Jecmenica et al. [72]	2011	Case report	2	Yes	Head, neck, chest, abdomen, pelvis, limbs injuries
Nwadiaro et al. [73]	2011	Retrospective study	485	No	Head, limbs injuries
Zhu et al. [74]	2011	Retrospective study	89	No	Limbs injuries
de Rome et al. [75]	2011	Retrospective study	212	No	Injury prevention strategies and technologies
Muggenthaler et al. [76]	2012	Case report	1	Yes	Head, chest, abdomen injuries
Zivkovic et al. [77]	2012	Retrospective study	381	Yes	Head, neck injuries
Heydari et al. [78]	2012	Retrospective study	542	Yes	Head, neck injuries
Júnior et al. [79]	2012	Retrospective study	367	No	Head, chest, abdomen injuries
Zhao et al. [5]	2012	Retrospective study	86	Yes	Head, chest, abdomen, limbs injuries
Carrasco et al. [80]	2012	Retrospective study	479	Yes	Head, chest, abdomen injuries
Bambach et al. [81]	2012	Retrospective study	1462	No	Head, chest, abdomen, limbs injuries
Zwolińska et al. [82]	2013	Retrospective study	242	No	Injury prevention strategies and technologies
Erdogan et al. [83]	2013	Retrospective study	226	No	Injury prevention strategies and technologies
Mosquera et al. [84]	2013	Retrospective study	17	Yes	Head, neck injuries
Mirza et al. [85]	2013	Retrospective study	581	Yes	Head, chest, pelvis injuries
Pircher et al. [86]	2013	Case report	2	Yes	Chest, abdomen injuries
Mohammadi et al. [87]	2013	Retrospective study	1564	Yes	Head injuries
Gioia et al. [88]	2013	Case report	1	Yes	Head, neck, chest, limbs injuries
Meredith et al. [89]	2014	Retrospective study	117	No	Injury prevention strategies and technologies
Edirisinghe et al. [90]	2014	Retrospective study	328	Yes	Head, neck, chest, abdomen, pelvis, limbs injuries
de Rome et al. [91]	2014	Retrospective study	117	No	Injury prevention strategies and technologies
Giustini et al. [92]	2014	Retrospective study	1821	No	Injury prevention strategies and technologies
Elachi et al. [93]	2014	Retrospective study	69	No	Head, limbs injuries
Hooten et al. [94]	2014	Review	/	No	Injury prevention strategies and technologies
Paryavi et al. [95]	2015	Retrospective study	2151	No	Limbs injuries
Lam et al. [96]	2015	Retrospective study	173	No	Head, neck injuries
Batista et al. [97]	2015	Retrospective study	3528	No	Head, neck, chest, abdomen, limbs injuries
Paryavi et al. [95]	2015	Retrospective study	759	No	Limbs injuries
Bambach et al. [98]	2015	Retrospective study	63	Yes	Head, neck injuries
Aikyo et al. [99]	2015	Experimental study	/	No	Injury prevention strategies and technologies
Chichom et al. [100]	2015	Retrospective study	621	No	Head, chest, abdomen, limbs injuries
Meredith et al. [101]	2016	Retrospective study	2151	No	Pelvis injuries
Ekmejian et al. [102]	2016	Review	/	No	Injury prevention strategies and technologies
Alexander et al. [103]	2016	Retrospective study	59	No	Limbs injuries
Liu et al. [104]	2016	Case report	3	Yes	Head, limbs injuries
Bedolla et al. [105]	2016	Retrospective study	78	No	Head, neck, chest, abdomen, limbs injuries
Lastfogel et al. [106]	2016	Retrospective study	85,689	No	Head, neck injuries
Erhardt et al. [107]	2016	Retrospective study	7051	No	Head injuries
Rice et al. [108]	2016	Retrospective study	/	No	Injury prevention strategies and technologies
Faduyile et al. [109]	2017	Retrospective study	156	Yes	Head, neck, chest, abdomen, pelvis, limbs injuries
Bell et al. [110]	2017	Case report	5	Yes	Head injuries
Albanese et al. [111]	2017	Retrospective study	62	No	Injury prevention strategies and technologies
Pelletti et al. [112]	2017	Retrospective study	8	Yes	Head, neck, chest, limbs injuries
Takeda et al. [2]	2017	Retrospective study	29	Yes	Head, neck, chest, abdomen injuries
Araujo et al. [113]	2017	Review	/	No	Injury prevention strategies and technologies
Osculati et al. [114]	2017	Case report	2	Yes	Head, neck injuries
Peng et al. [115]	2017	Review	/	No	Injury prevention strategies and technologies
Page et al. [116]	2018	Retrospective study	1061	No	Head, neck, chest, abdomen injuries
Ostby et al. [117]	2018	Case report	3	No	Neck, pelvis injuries
Saunders et al. [118]	2019	Prospective study	71	Yes	Head, neck, chest, abdomen, pelvis, limbs injuries
Meredith et al. [119]	2019	Experimental study	/	No	Injury prevention strategies and technologies
Gioia et al. [120]	2019	Case report	1	No	Head injuries
Bakovic et al. [121]	2019	Retrospective study	163	No	Head, chest, abdomen, limbs injuries
de Rome et al. [122]	2019	Experimental study	/	No	Injury prevention strategies and technologies
Madej et al. [123]	2019	Experimental study	/	No	Injury prevention strategies and technologies
Petetta et al. [124]	2020	Case report	1	No	Head, neck, chest, abdomen, pelvis injuries
Cheong et al. [125]	2020	Retrospective study	157	No	Head, chest, abdomen, limbs injuries
Jia et al. [126]	2020	Case report	1	Yes	Head, neck, chest, abdomen injuries
Medina et al. [127]	2020	Retrospective study	70	No	Head, neck injuries
Afquir et al. [128]	2020	Retrospective study	124	No	Injury prevention strategies and technologies
Lepard et al. [129]	2021	Review	/	No	Injury prevention strategies and technologies
Kleinertz et al. [130]	2021	Retrospective study	89	No	Head, chest, abdomen, limbs injuries
Saman et al. [131]	2021	Retrospective study	126	Yes	Head, neck, chest, abdomen, pelvis, limbs injuries
Cravez et al. [132]	2021	Retrospective study	561	No	Limbs injuries
Barron et al. [133]	2021	Retrospective study	419	No	Injury prevention strategies and technologies
Gentile et al. [134]	2021	Case report	1	Yes	Chest injuries
Martins et al. [135]	2021	Retrospective study	514	No	Head, limbs injuries
Anh NT et al. [1]	2021	Retrospective study	226	Yes	Head injuries
Tabary et al. [136]	2021	Review	/	No	Injury prevention strategies and technologies
Whyte et al. [137]	2022	Experimental study	/	No	Injury prevention strategies and technologies
Kent et al. [138]	2022	Retrospective study	1372	No	Head, neck, chest, limbs injuries
Lusetti et al. [139]	2022	Retrospective study	350	Yes	Head, neck, chest, abdomen, pelvis, limbs injuries
Emiogun et al. [4]	2022	Retrospective study	211	Yes	Head, neck, chest, abdomen, pelvis, limbs injuries
Tattoli et al. [9]	2022	Case report	1	Yes	Chest, abdomen, pelvis, limbs injuries
Ye et al. [140]	2022	Retrospective study	27	Yes	Chest, abdomen injuries
Urréchaga et al. [141]	2022	Review	/	No	Injury prevention strategies and technologies
Rauer et al. [142]	2023	Retrospective study	326	No	Limbs injuries
Liasidis [143]	2023	Retrospective study	22,855	No	Head, chest, abdomen, limbs injuries
Kleinertz et al. [144]	2023	Retrospective study	268	No	Head, chest, abdomen, limbs injuries
Meng et al. [145]	2023	Experimental study	/	No	Injury prevention strategies and technologies

One-hundred-eighteen papers fulfilled the inclusion criteria a) and b), as they presented findings from autopsy and clinical studies. Among these, 78 (66%) were retrospective studies, 33 (28%) were case reports, and 7 (6%) were prospective studies. The forensic studies, where autopsies were conducted were 63. The clinical studies conducted primarily on both living and deceased patients excluding autopsies were 55 and included the largest number of cases. Head injuries were the most extensively analyzed, appearing in the largest number of studies (89/118), followed by lesions to neck (73/118), chest and abdomen (chest 56/118; abdomen 44/118), pelvis (26/118), and limbs (55/118). The description and features of the lesions were extracted from each article and thoroughly discussed.

Twenty-four studies fulfilled the inclusion criteria (c), as they focus on injury prevention strategies and technologies. Among these, 10 (41%) were retrospective studies, 7 (29.5%) experimental studies, and 7 (29.5%) were reviews.

## Discussion

In traffic accidents involving motorcycles, injuries can occur in various regions of the body through different mechanisms. Given their severity, these injuries can lead to the victim’s fatality or, if not fatal, can offer valuable insights into the dynamics of the accident. The first five paragraphs discuss the characteristics of the injuries, based on a literature review, categorised by the body regions affected (head, neck, thorax-abdomen, pelvis and limbs). The sixth paragraph discusses the evaluation of injury prevention tests and technologies. Given the broad timespan of the literature review (1970–2023), each paragraph shows the chronological arrangement of cited sources corresponding to the respective topics under discussion. The final section examines elements critical to forensic assessment, including the role of injuries in causing fatalities and their importance in reconstructing accident dynamics.

### Head injuries

Head and facial injuries are the most frequent injuries observed in motorcycle accidents, with brain damage being the leading cause of death among motorcyclists [5, 34, 71, 131]. These injuries can result from various mechanisms. One major cause is the impact of victims’ head with other vehicles, fixed obstacles, or the ground when they are forcefully thrown after a collision due to the high inertia involved [71]. Additionally, brain injuries are often caused by deceleration forces, as the brain is not fixed, allowing it to relative movement within the skull. This can lead to various deceleration effects, such as multifocal vascular ruptures, cerebral concussion, or diffuse axonal damage [146]. Furthermore, injuries to the brainstem and pontomedullary region can result from significant movements like hyperextension, antero-flexion, and torsion of the head, caused by either direct contact trauma or acceleration-deceleration forces [147–149].

The most common head traumas observed in motorcycle accidents are concussions, followed by brain contusions or haemorrhages, facial and skull fractures [40, 143]. Wearing helmets prevents them: full-face helmets specifically reduce the incidence of brain contusions more effectively than the open-face type. However, the prevalence of skull fracture, subdural hematoma, and subarachnoid haemorrhage does not differ significantly between the two kinds of helmets [42]. Nonetheless, full-face integral crash helmets can also cause skull base fractures, as a portion of the impacting force is transmitted to the skull base through the chinstrap, involving the mandibular rami and condyles [15]. Moreover, heavier helmets are more likely to result in partial or complete ring fractures of the base of the skull when subjected to axial loading [25].

Brain injuries can also be linked to facial bone fractures. Studies have shown that fractures of the upper part of the face, such as the zygomatic and orbital bones, are more commonly associated with brain injuries than fractures of the lower part of the face, such as the mandibular bone [98, 150, 151].

Papers addressing skull fractures caused by direct impact have been distributed over the past 20 years [5, 34, 40, 42, 71, 98, 131]. Papers discussing severe ring fractures at the base of the skull associated with the use of full-face helmets originate from the 1990s [15, 25].

### Neck injuries

The most prevalent neck injuries due to motorcycle accidents include hemorrhage in the carotid sheath, subluxation in the occipital-atlanto-axial complex, hemorrhage in the muscles and triangles of the anterior neck, and damages along the vertebral artery [39].

The cervical spine region is the most affected part in case of fatal crashes [34]. Specifically, when the head undergoes hyperextension during a crash, the forces are transmitted through the cervical spine, leading to tissue damage [98]. The effectiveness of helmets in preventing cervical spine injuries remains a topic of debate. Some studies suggest their usefulness [116, 127], but Hitosugi et al. reported that the prevalence of cervical fractures was slightly higher in individuals wearing full-face helmets compared to those wearing the open-face type [42].

Regarding other helmet-related injuries, the helmet buckle can also cause fractures in the neck cartilage, primarily affecting the thyroid cartilage. The energy from the trauma results in the displacement of the helmet, and its buckle subsequently exerts pressure on the surrounding tissues. If the helmet buckle is positioned over the larynx at the moment of impact, it can exert enough force to potentially fracture the laryngeal cartilages [117].

Concerning neck vascular injuries, internal carotid artery dissection, though rare, has a high mortality rate after motorcycle accidents [124]. This type of injury may occur due to hyperextension and rotation of the neck, reflecting traction on the internal carotid artery as it crosses the transverse processes of the second and third cervical vertebrae. Another possible mechanism is abrupt full flexion of the neck, which can directly compress the internal carotid artery between the angle of the mandible and the upper cervical vertebrae [152]. Additionally, the pressure applied by the helmet strap on the soft tissues of the neck could also contribute to vascular damage [120]. A traumatic injury to the vertebral artery may determine death, especially in low-speed accidents, where the person may not immediately complain of specific symptoms after the accident. Instead, they may start feeling unwell hours or even days later, typically experiencing neurological symptoms like nausea, vomiting, and eventually leading to coma and death. The traumatic dissection of the vertebral artery causes cerebral infarction, followed by edema and compression of the brainstem. The vertebral artery is particularly susceptible to longitudinal stretch, and it can be hurt during sudden neck movements involving hyperextension and/or rotation. Therefore, in cases of delayed symptom presentation, forensic examination should take into account the possibility of vertebral artery dissection as a potential cause of death [61, 153].

Although rare, severe high-energy trauma can lead to the complete separation of neck tissues, even resulting in decapitation. The latter may be caused by different mechanisms, but it is often due to an impact against an immovable object [114]. Decapitation can also be attributed to the action of the lower edge of a full-face helmet during an incident. When a full-face helmet is worn during a traumatic event, the lower edge of the helmet can exert a significantly powerful force on the surrounding tissues, potentially leading to a complete cervical spine amputation [67, 88]. Additionally, instances of decapitation have been reported in the literature due to the interaction with motorcycle components. Ihama et al. reported a case in which a motorcyclist’s neck became entangled in a rotating motorcycle chain, resulting in complete decapitation [10].

All reported articles on neck injuries have been published within the last 20 years. Notably, certain articles focusing on helmet-related injuries [116, 127] and vertebral artery dissections [120, 124] encompass publications from the last 5 years.

### Chest and abdomen injuries

Injuries to the thorax and abdominal regions pose a significant risk of death due to severe blood loss, asphyxiation caused by thorax compression, and vertebral spine fractures [5, 33, 63, 121]. Common lesions in these areas include lung contusion and liver laceration, often occurring simultaneously with rib fractures [34, 81]. Impact during falls, especially between the left side of the abdomen and the end of the motorcycle handlebars, can cause splenic and pancreatic damages [44].

In high-velocity trauma, injury to the aorta is a typical and highly fatal occurrence. The ascending aorta is the most common affected segment, followed by the arch, thoracic, and abdominal sections [140]. Traumatic events in the ascending aorta or arch should be considered in cases of cardiac tamponade, aortic valve regurgitation, and myocardial contusion [84]. According to Richens et al., the aorta is subjected to various mechanical forces in anatomically vulnerable sites. Sudden deceleration can cause a stretching effect, leading to laceration of the isthmus as the ascending aorta and aortic arch are more mobile than the fixed distal descending part. Additionally, the aorta may rupture due to a sudden increase in blood pressure and entrapment between the anterior chest wall and the vertebral column [154]. The primary physiopathological mechanism of aortic laceration can be difficult to ascertain because of the diversity and complexity of crash scenarios, particularly when victims have been exposed to multiple external forces [112].

In major traumas, the thoracic spinal column may fracture. These injuries exhibit an “all or nothing” phenomenon, irrespective of the cause of the trauma, and tend to be more severe compared to similar lesions in the cervical region [127]. The thoracic spinal column may extensively suffer the trauma, or it remains intact with no damage. This effect is caused by the restraining effect of the ribs and sternum on the thoracic spine, making it less mobile. Consequently, if a force is powerful enough to break through a segment, the adjacent mobile segments are unable to absorb the remaining force, leading to displacement and significant injury to the spinal cord. Moreover, the thoracic canal provides slightly less space per segment compared to the cervical spine, favouring even slight displacement more dangerous [155].

Complete trunk severance cases are rare and typically associated with accidents involving high impact speeds. Muggenthaler et al. [76] documented a case of complete trunk severance resulting from a collision with a road signpost.

The articles mentioned in this paragraph have all been published in the last 20 years. Specifically, the articles addressing injuries to the aorta [112, 140] and spine [127] are from the last 5 years.

### Pelvis injuries

These are often attributed to contact with the motorcycle fuel tank during the collision [101]. These injuries can include fractures of the pelvic ring bones, damage to internal organs within the pelvic cavity, associated with pelvic haemorrhages of varying degrees, as well as lesions to the soft tissues of the lower abdomen, perineum, groin, or testicular area [9, 52].

Fuel tank injuries are typically experienced by the motorcycle driver, particularly in frontal collisions, when after impact, a sudden deceleration can propel the driver forward, colliding with the tank [71, 101, 156]. Interestingly, drivers who attempt to avoid a collision and topple over immediately before the impact are less likely to experience fuel tank injuries [52].

Even passengers can suffer groin injuries in accidents involving two riders on a motorcycle. In fact, passengers who are seated behind the driver often slide on the saddle, sometimes even onto the fuel tank, and then may hit the driver’s buttocks and back. However, the protection of the driver’s buttocks and back, can reduce the passengers’ risk of underbelly injuries [104].

The articles mentioned in this paragraph have all been published in the last 20 years. Specifically, some articles addressing fuel tank injuries [52, 101, 104] are from the last 5 years.

### Limb injuries

Motorcycle riders are particularly susceptible to limb injuries due to their heightened exposure to direct impacts [5]. Non-fatal limb injuries are the most frequent, encompassing ligamentous lesions, fractures, and dislocations [34, 38, 93]. These lesions occur when limbs become entrapped between the motorcycle and the ground or impact with fixed road signs or poles [48, 59, 97].

Regarding the upper limbs, the most common limb injuries are fractures of the shoulder, forearm, and hand [59, 97], reflecting the motorcyclist’s position, with flexed elbows being farthest from the impact point [132].

Forearm and hand injuries have been associated with lower mortality rates, because upper extremities act like a “crumple zone” when crash at highway speeds, protecting the head and neck region from direct impact in head-first hit damages in frontal crashes [95]. The distal portion of the upper extremity absorbs the energy of the collision, potentially reducing severe proximal trauma to the head and neck [12].

Hand lesions are more common in motorcycle drivers than in passengers, because during a collision, the driver instinctively locks their elbows and firmly holds onto the handlebars, redistributing the resulting force into the palm and metacarpal base. Thumb carpometacarpal joint injuries are particularly common due to the thumb’s position onto the handlebar grip making it more vulnerable to trauma [5, 103].

Concerning the lower limbs, the tibia is the most common site, followed by the proximal femur, particularly in lateral impact, patella and foot [20, 34, 125]. The heel is particularly susceptible with calcaneus fractures, Achilles tendon ruptures, and defects. The motorcycle’s lack of spoke guards or poorly designed guards favours the entrapment of the pillion passenger’s heel between the spokes and the frames, resulting in crushing and grinding injuries from the continuously rotating wheels [30, 74].

The articles cited in this paragraph cover a wide range of time periods, from the 1970s to the 1990s [12, 26, 30] to the 2020s [125, 132]. This temporal variety is evident in articles discussing both upper and lower limb injuries.

### Injury prevention strategies and technologies

The topic of injury prevention strategies and technologies has gained increasing interest in recent years. In fact, the articles mentioned in this paragraph have all been published since 2009, and a substantial number of them are very recent, with publication dates within the last five years [113, 115, 119, 122, 123, 128, 129, 133, 136, 137, 141, 145].

Injury prevention strategies and technologies for motorcyclists have focused on two main aspects. Firstly, helmets are used to primarily protect the head, an area highly correlated with increased mortality rates. Secondly, protective clothing and wearable devices are employed to shield the remaining parts of the body.

The mandatory use of motorcycle helmets for both drivers and adult passengers has become a global legal requirement, with few discrepancies in regulations across the world. However, variations persist, particularly regarding restrictions on transporting child passengers in Asia and Africa, despite the prevalence of motorcycle use in these continents [129, 157].

It is well known that motorcyclists wearing helmets tend to experience less frequent and severe episodes of head and facial injuries. Helmets have demonstrated effectiveness in mitigating and preventing traumatic brain injuries in motorcycle accidents, particularly during impacts with large energy-absorbing surfaces, for example when the head collides with the ground or the surface of a car body. However, the protective capacity of helmets is constrained when the materials they are made of surpass their tolerance thresholds, as in high-speed impacts or direct trauma with objects presenting limited surface areas, such as light poles, trees, or angular parts of the vehicle [131, 133, 139, 143].

Modern helmets are designed to have a rigid polycarbonate outer shell with a firmly placed energy-absorbing liner, while a soft expanded polystyrene or polyurethane foam padding forms the innermost layer.

Helmet damage can range from subtle blemishes to obvious defects, and their careful examination can provide valuable information about the accident dynamics and cranial injuries sustained by the motorcyclist. Conventional helmet damage assessment involves the manual removal of each layer, a process prone to oversight and additional damaging. To reduce these risks, the use of computed tomography scanning of the helmet has been suggested. This method allows precise delineation of damage within each layer, including breaks in the outer shell or compression in the inner layers. The radiological examination can also facilitate measurements of the thickness and density of the foam and can detect bloodstains, confirming that the helmet was worn by the motorcyclist at the time of impact [158].

While full-face helmets provide comprehensive protection, open-face and half-cover helmets, although less secure, are still in use [42, 145]. Recent studies suggest that full-face helmets offer better protection against head and face injuries, but may be less effective in preventing neck injuries and skull base fractures [136, 145]. Notably, motorcycle full-face helmet visors, while not designed for energy absorption, redirect mid-face impact forces to the upper and lower face, enhancing protection [145]. Despite the overall recommendation for full-face helmets due to their reduced mortality and injury probability, their usage is often limited due to discomfort, particularly in subtropical and tropical climates where most motorcyclists reside [141].

Despite the universally recognized head protection helmets offer, a debated issue remains concerning a potential increase in the risk of cervical spine injuries due to increased hyperflexion-hyperextension movements induced by the helmet’s lower edge [15, 117, 133]. Some studies suggest that helmet use reduces the risk of cervical spinal cord injuries during motorcycle crashes [116, 127]. However, Hitosugi et al. [42] reported a prevalence of cervical fractures wearing full-face helmets. The literature is inconclusive, suggesting that helmet use does not directly increase spinal injuries, but may be less effective in preventing severe cervical injuries compared to cranial injuries [94, 108]. Nevertheless, the present review indicates that fatal skull base ring fractures associated with the use of full-face helmets have been reported in articles dating back to the 1990s [15, 25]. This reinforces the notion that contemporary helmets are effective in preventing more severe spinal injuries. On this point, advancements in helmet technology have recently introduced airbag-equipped helmets, representing a novel class of protective headgear. These helmets, equipped with inflatable structures, have demonstrated a reduction in concentrated impact force to the lower- or mid-face region, resulting in decreased head rotation and brain strain. Contrary to conventional helmets, these devices only deploy when necessary, preserving the field of vision and ventilation during normal use [145].

In injury prevention, wearing a helmet is not only crucial but equally important is the correct method of wearing it. Proper helmet fixation and correct strap positioning are essential for effective prevention of head and neck injuries. Loose helmet straps can cause ejection or compromise anterior neck structures in high-impact crashes [136]. Moreover, helmeted individuals are significantly less likely to sustain shoulder fractures, suggesting a protective effect of helmets. A possible explanation is that energy transmission through the helmet and neck flexion during the impact may shield the proximal humerus and shoulder girdle [132].

The current regulation from January 2021 is ECE 22.06, which introduces new test procedures for the production and assessment of next-generation helmets. These include a rotational acceleration test to assess oblique impacts, linear impact tests for both high and low energy impacts, testing of retention systems and new assessments for helmet visors to measure impact resistance against small objects thrown at high speed. In addition, ECE 22.06 requires a test to assess helmet stability on the head. While the previous standard, ECE 22.05, simulated the risk of “roll-off”, where the helmet rotates forward and disengages from the rider’s head on impact, ECE 22.06 extends the assessment to the possibility of backward rotation, potentially exposing the rider’s forehead or neck after impact. This evaluation includes the open chin guard tear test, which ensures complete detachment of the chin guard to reduce the risk of neck injury. ECE regulations also assess the point of impact on the helmet during a crash. While ECE 22.05 identified six primary impact points (front, top, back, sides, and chin), ECE 22.06 extends the evaluation to include 12 additional intermediate impact points along the mid-lines [159].

Concerning protective clothing for motorcyclists, epidemiological observations highlight the vulnerability of light motorcycle drivers compared to heavy motorcycle users. Light motorcycle drivers, often not wearing protective clothing, are statistically more involved in road accidents, as these motorcycles are commonly used for work and transport [83]. Technical clothing such as jackets, pants, shoes, and gloves has shown effectiveness in preventing soft-tissue injuries, particularly open wounds, but has no significant impact on systemic injuries or fractures [75, 83].

The usage of protective clothing reduces the probability of upper (shoulder and elbow) and lower limb (buttock and thigh) skin injuries, while providing less effective protection for the chest, abdomen, back, and groin [91]. However, the use of motorcycle protective clothing in warm seasons may impair cognitive and psychophysical functions, potentially affecting riding performance and safety due to body overheating [82, 91].

Given the limitations of protective clothing, specific impact protection technologies have been developed for areas of the body where protection is limited, such as the back and abdomen [91]. Nevertheless, their adoption remains uncommon among on-road motorcyclists, and there are no laws governing the usage of such protective measures [139].

Protective clothing cannot entirely prevent limb fractures during falls, especially severe ones affecting the proximal femur in lateral impacts. Hard hip protectors, designed to cover the greater trochanter, effectively absorb and redirect impact energy away from vulnerable areas, preventing direct hip fractures [64]. Studies on limb injuries cover several decades, from the 1970s through the 1990s [12, 26, 30] to the 2020s [125, 132]. During this wide timeframe, to the best of our knowledge, no effective protective device suitable to prevent limb bone or soft tissue injuries was developed. Indeed, while current protective equipment is effective in preventing skin injuries, it still has significant limitations in preventing more severe damage [75, 83].

Back protectors, initially designed for racing sports, have shown effectiveness in reducing back cutaneous injuries but are limited in protecting against spinal injuries caused by bending and torsional forces [102, 128]. The integration of hard-shell or airbag technologies into back protectors may enhance their effectiveness in preventing serious spinal injuries [92].

Recent developments include rider-worn pelvic protection devices designed to reduce the risk of injury from contact with the motorcycle fuel tank during a crash. Simulation studies suggest potential benefits in the absorption and distribution of impact energy, but the understanding of pelvic biomechanics under anteroposterior loading is currently limited [137].

The effective prevention of trunk injuries remains a subject of ongoing study. The substantial number of recently published studies examining these injuries, particularly in relation to aorta laceration [112, 140], spinal fractures [127], and fuel tank injuries [52, 101, 104], underscores the persistent challenge posed by these injuries.

### Injuries evaluation in the reconstruction of the crash dynamics

The evaluation of injuries for analyzing the dynamics of the accident is of forensic significance in criminal, civil, or insurance cases, since the results of medical examinations or autopsies enhance the information gathered from on-site inspections and circumstantial data. Fatal injuries typically result from direct impacts on the body during accidents involving other vehicles, the ground, or fixed obstacles. In many cases, multiple impacts usually involve an initial collision with objects, followed by a secondary impact with the ground. Furthermore, the subjects may sustain tertiary injuries due to being struck by vehicles or colliding with fixed obstacles such as road poles, walls, or barriers [81, 131].

In fatal cases from direct impacts the primary cause of death is a neurogenic shock due to a direct head impact [40, 71]. The site of cranial blunt trauma can be determined by combining autopsy findings, typically skull fractures and intracranial hemorrhages, with data collected from the accident scene. Therefore, it is crucial to establish whether the victim was wearing a helmet and, in such instances, to have the ability to examine it. Locating the break points on the surface of the helmet can provide crucial information regarding the precise point of impact.

It is also paramount to assess the helmet’s fit on the subject, by measuring the length of the buckle to assess its suitability for the subject’s facial and neck proportions, given that excessive helmet excursion during a crash can potentially led to laryngeal cartilage fracture which can be better identified by radiological studies such as computed tomography.

Direct trauma to the thorax and abdomen can result in internal thoracoabdominal organ lacerations, leading to fatal hemorrhagic shock. Such trauma can occur due to contact with the ground or components of the motorcycle, such as its handlebars [5, 33, 44, 63, 121]. At autopsy, if that kind of vehicle collision dynamics is suspected, bruising patterns that mimic the shape of handlebar components should externally be sought. During the examination of the body, consideration should be given to whether the victim was wearing protective motorcycle clothing. This factor can potentially mitigate skin injuries, particularly on the limbs, making them less apparent on external inspection.

Fatal injuries can be attributed also to the forces of acceleration and deceleration resulting from sudden, unrestricted body movements, which induce compressive, tensile, and shear strains of the tissue. Specifically, neck hyperextension can damage the brainstem and cervical region, presenting at autopsy as brain hemorrhages without cranial fractures, fractures of the cervical vertebrae, soft tissue hemorrhages, or carotid artery dissection. This vessel is particularly susceptible to hyperextension movements as the vascular wall can undergo stretching and tearing [34, 98, 124]. Even in cases of low-energy trauma where the subject deceases some days after the accident, suspicion should fall on vertebral artery dissection. This vessel is notably vulnerable to longitudinal stretching and can rupture even in mild hyperextension or neck rotation [61, 153]. A meticulous macroscopic and microscopic examination of the vertebral artery is mandatory when symptoms such as nausea and vomit were reported in clinical record.

In the thoracoabdominal region acceleration and deceleration forces can cause fatal injuries especially in high-energy trauma with the involvement of the aorta, that is highly susceptible to stretching and tearing, primarily in its more mobile segments, such as the isthmus and arch [140, 154], resulting in hemorrhagic shock without significant direct trauma indicators. A violent hyperextension of the thoracic spine can lead to severe vertebral fractures and spinal cord damage due to the limited mobility of this portion, which is firmly anchored to the ribs and sternum [127, 155].

In the forensic medical evaluation of a motorcycle accident, one of the most challenging and pertinent issues is distinguishing the driver from the passenger. In literature, it is reported that drivers are predominantly male, while passengers are female [139]. When analyzing motorcyclist fatalities by age, a notable spike is observed among subjects aged between 21 and 30 years. This trend can likely be attributed to a lack of riding experience and impulsive behavior among teens and younger adults [131]. Additionally, a higher prevalence of fatal crashes occurring at night and on weekends has been reported, with positive toxicology tests for alcohol and drugs for both drivers and passengers [139]. In most cases, it has been observed that drivers were wearing helmets at the time of the accident, whereas passengers more commonly did not wear helmets, thereby increasing the likelihood of sustaining severe traumatic brain injuries [143]. Nevertheless, these are general epidemiological data obtained from literature review, and cannot be used to discriminate between the passenger and the driver in individual cases.

The injuries sustained can provide some useful information. The ejection dynamics differ for drivers and passengers due to variations in their initial positions and postures.

The rider’s distinctive attitude is to hold the handlebars while driving, thus establishing a direct connection between himself and the motorcycle. In case of a collision, this connection can prevent the rider from being ejected from the motorcycle, thus increasing the risk of sustaining crushing or burning injuries as a result of impact with various parts of the motorcycle. Such incidents are more likely to occur in low energy collisions, as in high energy collisions the rider is typically thrown and ejected at a distance due to the significant inertial forces, preventing them from remaining anchored to the vehicle [71].

Conversely, pillion passengers have different options for support. They can either grasp the bar attached to the tail of the motorcycle or hold onto the driver’s haunches in front of them. However, due to their less stable position, pillion passengers are typically propelled higher into the air during the accident, resulting in a longer fall to the ground from a greater distance [71].

Some specific lesions may serve as distinguishing factors between the driver and the passenger involved. Passengers can suffer groin injuries, usually mild and caused by impact with the driver’s buttocks and back [104]. Furthermore, the passenger’s heel can become entrapped between the spokes and the rear wheel frame, resulting in crushing and grinding injuries [30, 74]. In drivers, lesions to the palm of the hand and the thumb carpometacarpal joints are more prevalent. This is because, during a collision, drivers tend to instinctively lock their elbows and grip the handlebars tightly [5, 71, 103]. Pelvic damage and fractures, called fuel tank injuries, tend to be associated with the driver’s impact against the fuel tank in frontal crashes where the sudden deceleration leads to the body sliding forward making contact with the fuel tank [71, 101, 156].

Nevertheless, the reconstruction remains a challenging task due to the intrinsic complexity of accident dynamics. This procedure entails a multidisciplinary approach, as demonstrated in many specific cases, mostly for the identification of the manner of death, consisting in conducting interviews with individuals directly involved in or witnessing the incident, performing mechanical and engineering examinations (i.e., a kinematic analysis) and procuring documented visual evidence of the occurrence [160, 161]. In cases involving motorcycle accidents, recorded images of the event can be sourced from various means, including surveillance cameras, helmet-mounted cameras worn by riders, and dashboard-mounted cameras in the vehicles participating in the accident.

A critical analysis of findings collected at accident scenes, including skid marks and the resting positions of vehicles, combined with the forensic evaluation of injuries in the light of developments in safety devices, addresses medical-legal responses based on scientific evidence.

## Conclusion

This review highlights that, in addition to injuries commonly associated with motorcycle accidents, such as head injuries, there are also unique lesions linked to the seating position of the passenger and the specific nature of the trauma. Given the considerable variability in real-life accident scenarios and the frequent lack of comprehensive crash details, the forensic assessment of injury distribution could be a support to aid in reconstructing the dynamics of traffic accidents and to define cause of death in the crash fatalities.

## Limits

While the PRISMA guidelines are widely accepted standards for conducting systematic reviews in various fields, the application of these guidelines in the present forensic pathology article presented specific challenges. In particular, the data of interest consisted primarily of qualitative information, involving descriptions of injuries and interpretations of accident dynamics, rather than numerical measurements or quantifiable data points. As a result, qualitative data inherently exhibited high variability, which made it challenging to standardise or homogenise in order to draw coherent and general conclusions within the field of forensic pathology.

## Data Availability

Not applicable.
